# Multi-omics approaches for in-depth understanding of therapeutic mechanism for Traditional Chinese Medicine

**DOI:** 10.3389/fphar.2022.1031051

**Published:** 2022-11-25

**Authors:** Xue Zhu, Qi Yao, Pengshuo Yang, Dan Zhao, Ronghua Yang, Hong Bai, Kang Ning

**Affiliations:** ^1^ Key Laboratory of Molecular Biophysics of the Ministry of Education, Hubei Key Laboratory of Bioinformatics and Molecular-imaging, Center of AI Biology, Department of Bioinformatics and Systems Biology, College of Life Science and Technology, Huazhong University of Science and Technology, Wuhan, Hubei, China; ^2^ Dovetree Synbio Company Limited, Shenyang, China

**Keywords:** traditional Chinese medicine, multi-omics approaches, quality control, pharmacodynamic effects, network pharmacology analysis, TCM databases

## Abstract

Traditional Chinese Medicine (TCM) is extensively utilized in clinical practice due to its therapeutic and preventative treatments for various diseases. With the development of high-throughput sequencing and systems biology, TCM research was transformed from traditional experiment-based approaches to a combination of experiment-based and omics-based approaches. Numerous academics have explored the therapeutic mechanism of TCM formula by omics approaches, shifting TCM research from the “one-target, one-drug” to “multi-targets, multi-components” paradigm, which has greatly boosted the digitalization and internationalization of TCM. In this review, we concentrated on multi-omics approaches in principles and applications to gain a better understanding of TCM formulas against various diseases from several aspects. We first summarized frequently used TCM quality assessment methods, and suggested that incorporating both chemical and biological ingredients analytical methods could lead to a more comprehensive assessment of TCM. Secondly, we emphasized the significance of multi-omics approaches in deciphering the therapeutic mechanism of TCM formulas. Thirdly, we focused on TCM network analysis, which plays a vital role in TCM-diseases interaction, and serves for new drug discovery. Finally, as an essential source for storing multi-omics data, we evaluated and compared several TCM databases in terms of completeness and reliability. In summary, multi-omics approaches have infiltrated many aspects of TCM research. With the accumulation of omics data and data-mining resources, deeper understandings of the therapeutic mechanism of TCM have been acquired or will be gained in the future.

## Introduction

Traditional Chinese Medicine (TCM) has a long history of treating a variety of diseases around the world (Lan et al., 2015; Xiang et al., 2019; Runfeng et al., 2020). The most critical concerns of TCM are the quality of TCM formulas, as well as the effectiveness of TCM formulas, both of which have been extensively studied by researchers and physicians (Jia et al., 2017; Xin et al., 2018a; Xin et al., 2018b; Bai et al., 2019; Xiang et al., 2019; Ding et al., 2020; Liu et al., 2020; Chandrasekara et al., 2021). Western medications are typically prescribed individually for a specific effect with a single component. In contrast, classical TCM formulas, following TCM theory and the principle of “King, Vassal, Assistant, and Delivery servant,” are comprehensive systems with “multiple ingredients, multiple targets, and multiple pathways” to exert the synergistic effect in the prevention and treatment of diseases (Yi and Chang, 2004; Wang and Zhang, 2017). Their complexity in terms of components (especially for these structurally similar components), metabolites, and bioactivities, also hampered research into the therapeutic mechanism of TCM in treating various diseases. With the development of high-throughput sequencing technology, system biology-driven omics approaches meet the needs of TCM research, which could be applied to decoding complex components, targets, and drug-disease interactions (Subramanian et al., 2020). The multi-omics approaches refer to a series of research methods based on high-throughput analysis in modern biological research systems, which mainly include genomics, transcriptomics, metagenomics, metabolomics, epigenomics, and proteomics (Olivier et al., 2019; Subramanian et al., 2020). From the perspective of omics approaches, TCM could be considered as a combination of small molecules. Thus, a single TCM could treat multiple diseases by combining the small molecules with their respective gene/protein targets. After thousands of years of therapeutic practice in China, researchers have found a vast number of TCM formulas that are utilized for treating numerous diseases based on multi-omics approaches and clinical experiments, contributing to new drug discovery and therapeutic mechanisms (Buriani et al., 2012; Olivier et al., 2019; Guo et al., 2020), as well as advancing TCM towards precision medicine (Olivier et al., 2019).

While TCM has been extensively used in the treatment of various kinds of acute and chronic diseases worldwide (Bai et al., 2015; Kim et al., 2010; Miryala et al., 2018; Yang M. and Lao), several limitations have hindered the development of TCM research, including TCM formula quality control, multi-omics approaches on TCM analysis, and data resources for TCM research. Firstly, the quality control of TCM formulas is highly contentious (Yao et al., 2022). Thus, much attention should be paid to its ingredients and production process to ensure its safety and efficacy. Only if the ingredients of TCM formula are safe and lawful, TCM could exert its power in clinics worldwide, laying the groundwork for further studying the therapeutic mechanism, bioactive compounds, targets, and drug discovery to benefit humans. Moreover, TCM research is primarily experience-based and experiment-based methods, in which the small molecules of TCM and the specific targets for the treatment of diseases have not been thoroughly investigated, which impedes the modernization of TCM. Furthermore, the regulating principles of TCM formulas in human body behind the treatment of various diseases remain largely unclear.

Luckily, with the development of sequencing technology and system biology-driven omics approaches, TCM research has also advanced (Zhang et al., 2019b). The mechanisms of TCM and side-effects of TCM formulas were further deciphered at the molecular level (Buriani et al., 2012; Cai et al., 2018; Subramanian et al., 2020), leading to new therapeutic directions (Yang and Lao, 2019; Guo et al., 2020). These efforts are potentially accelerating TCM towards modernization (Qiu, 2007), and enhancing the applicability of the TCM formulas for personalized treatment (Aronson and Rehm, 2015; Jameson and Longo, 2015; Miryala et al., 2018). The concept of network pharmacology analysis is proposed to ascertain which bioactive compounds and targets are effective in treating various diseases. This approach entails deciphering a “compound-protein/gene-disease” network for TCM formulas and elucidating the regulatory principles of small molecules in a high-throughput manner. Furthermore, TCM-associated databases have been developed to meet the needs of TCM research, serving as a repository for TCM formula, herbal ingredients, bioactive compounds, targets, and TCM-disease interactions, and feedback for more comprehensive analysis of the network pharmacology analysis and omics research. These efforts would accelerate the internationalization and digitization of TCM formulas. However, to digitalize all TCM-related resources and fully excavate these resources for deeper understanding of TCM materials and formulas, the help of bioinformaticians would be important. In contrast, bioinformaticians need to better understand TCM research (Gómez-López et al., 2019).

**FIGURE 1:**
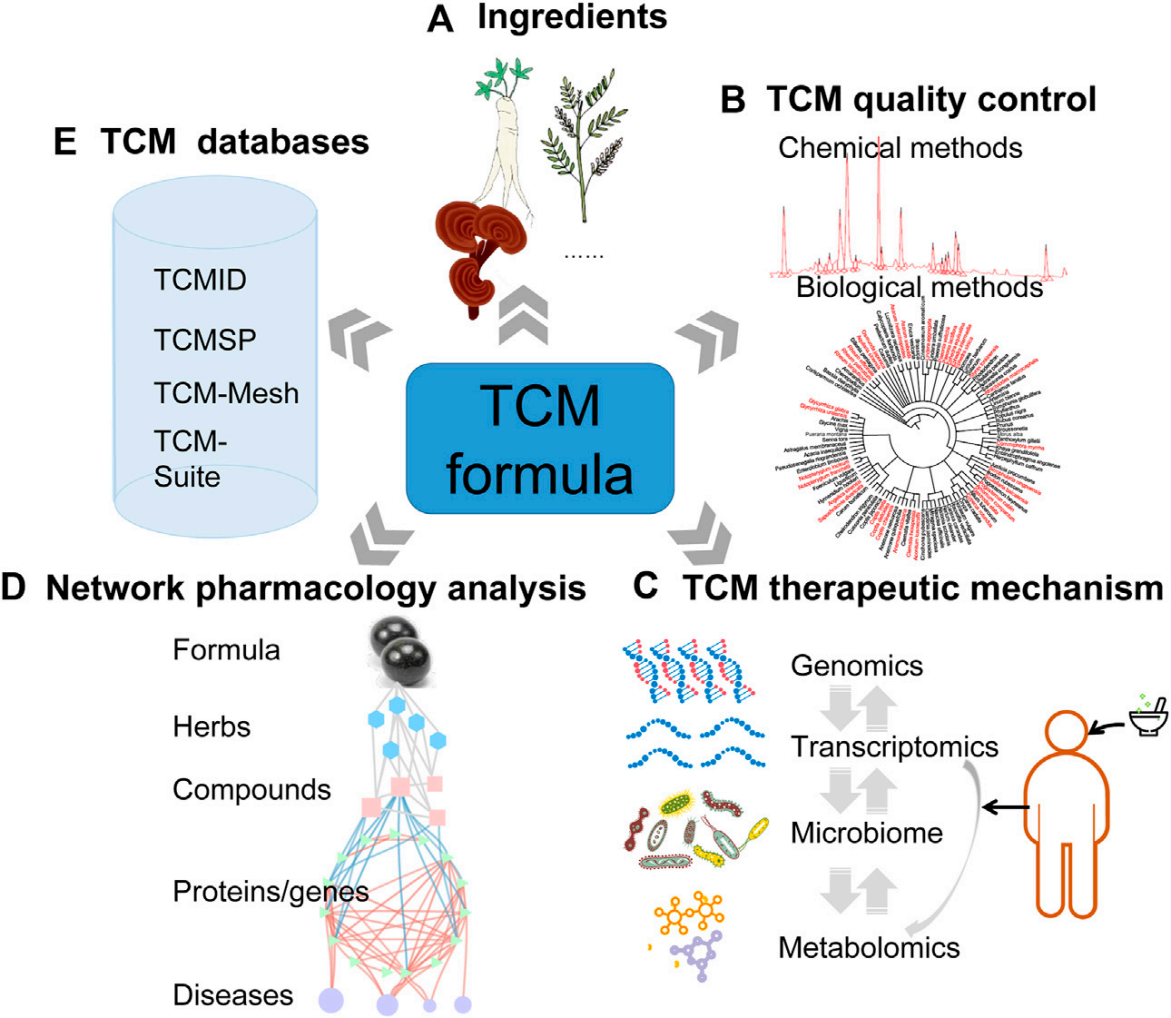
Systems biology-driven omics approaches have boosted TCM research from multiple perspectives. **(A)** Prescribed ingredients of TCM formula recorded in Chinese Pharmacopoeia (2020 edition). **(B)** The TCM quality control (fingerprint-based chemical ingredients analysis methods and genomics-related biological ingredients analysis methods). **(C)** Multi-omics approaches including genomics, transcriptomics, microbiome, and metabolomics could be utilized for investigating the therapeutic mechanism of the TCM formula for treating various diseases. **(D)** Network pharmacology analysis for screening the bioactive compounds and potential targets for TCM formulas in treating various diseases. **(E)** The databases for TCM research. The current databases serve as a link across TCM formulas, ingredients, bioactive compounds, targets (proteins/genes), and various diseases.

In this review, we have reviewed and summarized the recent progress of multi-omics approaches in deciphering the mechanism of TCM against various diseases (Figure 1). We first discussed the currently widely used quality control methods of the TCM formula. Chemical and biological ingredients (Figure 1A) are both indispensable components for TCM, and we could assess TCM quality by detecting these ingredients using fingerprint-based chemical methods and DNA-sequencing-based biological methods (Figure 1B). Secondly, we emphasized the importance of integrating multi-omics in decoding the mechanism of TCM formulas in treating various diseases (Figure 1C). Thirdly, we also discussed the interaction of bioactive compounds and disease targets from the perspective of network analysis (Figure 1D). Finally, we compared different TCM databases based on their basic properties and search results (Figure 1E). These initiatives may help unravel the “black box” of the therapeutic mechanisms of the TCM formula, provide the best practices of contemporary bioinformatics analysis for TCM formulas, as well as contribute to the TCM toward digitalization, internationalization, and precision medicine. Of course, to translate these advances and fully excavate these resources, the routine clinical practice needs a bioinformatician with good training in clinics, which will help multi-omics approaches and datasets be of importance in better understanding the TCM towards precision therapeutics.

## Omics approaches for TCM formula quality control: Biological and chemical ingredient analysis

Omics approaches, including fingerprint-based chemical ingredients analytical methods and DNA-sequencing-based biological ingredients analytical methods (related to genomics approaches), are essential for the quality assessment of TCM formulas. With the popularity of TCM, its quality control methods have become an important research topic to ensure its safety and effectiveness and thus promote the modernization and internationalization of TCM (Cheng et al., 2014; Baing et al., 2019; Yao et al., 2022). Although standards for the quality control of botanical drugs, processed products, TCM extracts, and TCM formulas have been documented in the Chinese Pharmacopoeia (ChP, 2020 edition) (Xu et al., 2021), the methods recorded in ChP are capable of discriminating substances from morphological, chemical, and physical characteristics, they are not precise enough to distinguish the ingredients from genetically related or hybrid species (Cheng et al., 2014).

### The methods for chemical ingredient analysis

Currently, the most extensively utilized methods for TCM quality control are fingerprint-based methods (Cheng et al., 2014; Bai et al., 2019), such as thin-layer chromatographic scanning (TLCS), high-performance liquid chromatography (HPLC), gas chromatography (GC), and mass spectrometry (MS), which are focused on the bioactive compounds of the TCM formula. Among them, HPLC is characterized by high separation efficiency, high selectivity, high detection sensitivity, and rapid analysis, which is broadly applicated and usually coupled with other chromatographic methods in assessing the chemical ingredients of TCM formula (Heinisch and Rocca, 2009). For example, liquid chromatography-mass spectrometry (LC-MS)-based metabolic method has been utilized to detect the bioactive compounds of Kaixin Powder (KXP) in treating mentally depressed diseases (Zhu et al., 2010). They have found several bioactive compounds: ginsenosides, 3,6′-disinapoyl sucrose, α/β-asarone, and pachymic acid for *Ginseng Radix* et Rhizoma, *Polygalae Radix*, *Acori Tatarinowii* Rhizoma, and *Poria* in KXP based on LC-MS, respectively (Zhu et al., 2010). Apart from these five compounds, Wang et al. (2021), have firstly found two novel bioactive compounds of KXP (dehydrotrametenolic and dehydrotumulosic acids) from KXP using HPLC-DAD and HPLC-QTOF-MS/MS. These efforts have also resulted in the widespread use of chromatographic methods for TCM formula quality assessment (Liu et al., 2016b; Fan et al., 2021; Zeng et al., 2021).

### The methods for biological ingredient analysis

On the other hand, biological ingredients are also indispensable components for TCM formulas, which could be identified through DNA barcoding-based approach. Out of the plenty of DNA barcodes (Table 1), ITS2, *trnL*, and *psbA-trnH* are the most frequently used herbal DNA barcodes, while COI is a well-known standard DNA barcode for animal materials detection (Coghlan et al., 2012; Li et al., 2018; Zhang et al., 2019a). Numerous studies have also used these representative barcodes to assess the biological ingredients of TCM formulas by detecting their prescribed species using high-throughput sequencing, which has detected 44.4%–100% of the prescribed ingredients of TCM formulas, suggesting the high universality, sensitivity, and specificity of this approach (Cheng et al., 2014; Jia et al., 2017; Joseph and Pe’er, 2021; Li et al., 2018; Xin et al., 2018a; Yao et al., 2022). In addition, DNA barcoding could also be applied to detect whether the prescribed ingredients are replaced by similar ingredients, such as the *Arisaematis Rhizoma* is replaced by *Pinellia pedatisecta* in the Ruyi Jinhuang Powder (Li et al., 2018). This approach could also be applied in auditing whether TCM formulas contain derivatives of protected species, especially for these endangered, trade-restricted species of plants and animals (Coghlan et al., 2012; Chen et al., 2015), which could promote the protection of wild species. Taken together, the DNA-barcoding approach innovatively identifies the prescribed ingredients of TCM at molecular level. It breaks away from the traditional morphological identification methods that heavily rely on long-term experience or experiment results, providing the “gene identity card” for TCM materials, and contributing to the standardization of the TCM industry.

**TABLE 1:** The candidate DNA barcodes for identifying ingredients of TCM formulas.

DNA barcodes	Abbreviation	Gene type	Characteristic	Application	Sensitivity (%)	References
Internal transcribed spacer 1	ITS1	Nucleus gene	High inter-specific and intra-specific divergence, but poor PCR amplification	Plant barcode	52∼88.2	(Luo K. et al., 2010; Chen et al., 2010)
Internal transcribed spacer 2	ITS2		High inter-specific and intra-specific divergence with high species identification ability	Core plant barcode	92.7	Chen et al. (2010)
*psbA-trnH*	*psbA-trnH*	Chloro-plast gene	High variation and species identification ability	Core plant barcode	75∼100	(Jia et al., 2017; Zhang P. et al., 2019a)
Chloroplast genome trnL (UAA) intron	*trnL*		Short fragment; multiple internal conserved primer sites and easily amplified in heavily degraded DNA samples; not applicable for fungi	Plant barcode	44.4∼100	(Coghlan et al., 2012; Cheng et al., 2014; Yao Q. et al., 2022)
maturase K	*matK*		High variation and species identification ability, but lacks universally conserved primer-binding sites	Plant barcode	∼72	(Group, 2009; DUNNING and SAVOLAINEN, 2010; Chen et al., 2010)
*rbcL*	*rbcL*		High discriminatory power in the cryptogam; ambiguous species identification	Plant barcode	35.6∼87	(Chen et al., 2010; Kolter and Gemeinholzer, 2021)
*ropB*	*ropB*		Low variation and low identification ability	Plant barcode	<50	(Group, 2009; Chen et al., 2010)
Cytochrome oxidase I	COI	Mitoch-ondrial gene	Standard DNA barcode for animals	Core animal barcode	∼90	(Vences et al., 2005; Zhang P. et al., 2019a)

However, there are also some limitations and challenges in fully utilizing the methods for TCM quality assessment. Firstly, the methods for chemical ingredients analysis were unable to discriminate the ingredients with comparable or unambiguous peaks (Wolfender et al., 2003). Secondly, fixating on chemical ingredients overlooks the biological characteristics that are also indispensable components of TCM formula (Cheng et al., 2014; Bai et al., 2015; Bai et al., 2019). Thirdly, though DNA barcoding-based approaches, the prescribed ingredients (recorded in ChP) of TCM formula could be detected (Table 1), they are unable to detect all prescribed ingredients of TCM formula. For example, *trnL* is incompatible with detecting fungal ingredients in TCM formulas (Cheng et al., 2014), and *rbcL* is a well-characterized barcode in GenBank, but this barcode is limited in identifying species from the same genera (Newmaster et al., 2006; Besse et al., 2021). Thus, more DNA barcode candidates should be considered for TCM biological ingredients assessment. Fourthly, the referenced prescribed species in the GeneBank are not enough for species identification. Further studies should take more databases into consideration, such as the DDBJ, EMBL, and PDB databases (Coghlan et al., 2012), TCM-suite (Yang et al., 2022), as well as tcmbarcode (Chen et al., 2014), and other databases. Finally, the genomics-based DNA barcoding approach could not detect the mineral ingredients. At the same time, the fingerprint-based chemical methods could overcome this drawback, and thus combining the methods for chemical ingredients analysis and the biological ingredients analysis could yield a more comprehensive and reliable assessment for TCM. Collectively, these efforts will benefit the safety issues of TCM formulas and accelerate the digitalized management process and internationalization of the TCM industry.

## Omics techniques for understanding the therapeutic mechanism of TCM in treating various diseases

**FIGURE 2:**
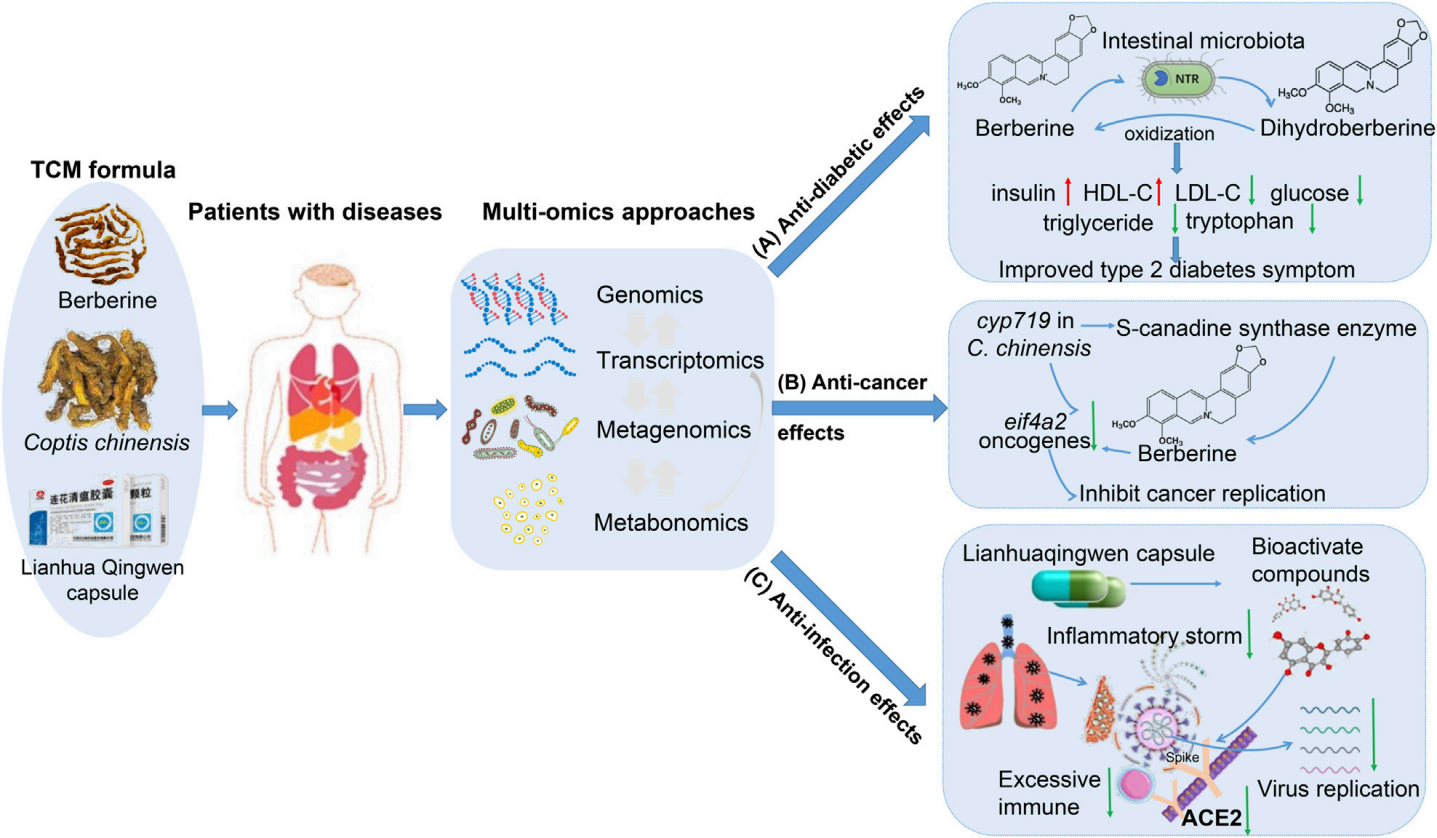
Multi-omics approaches are applicable for investigating the therapeutic mechanism of TCM in treating diseases. In this review, we used berberine, *C. chinensis* Franch. (Ranunculaceae), and Lianhua qingwen capuse as examples to illustrate the critical role of multi-omics approaches in elucidating the therapeutic mechanism of TCM. **(A)** Multi-omics analysis for berberine in treating type 2 diabetes mellitus. In this case study, intestinal microbes converted berberine as host absorbable molecule dihydroberberine to improve diabetes. NTR: nitroreductase. **(B)** Multi-omics analysis of *C*. *chinensis* Franch. (Ranunculaceae) for anti-cancer effect. Through multi-omics analysis, the functional genes (i.e., *cyp719*) and bioactive compounds (i.e., berberine) of *C. chinensis* Franch. (Ranunculaceae) have suppressed the eukaryotic translation initiation factor 4A isoform 2 (*eif4a2*) expression, and thus blocked cancer replication. **(C)** Multi-omics analysis for the potential therapeutic mechanism of Lianhua Qingwen capsule in COVID-19 patients. Through multi-omics analysis, the bioactive compounds of Lianhua Qingwen capsule could inhibit the combination of the viral target Spike and human target ACE2, thus preventing the viruses’ invasion and attachment to host cells and inhibiting the virus replication and pro-inflammatory cytokine levels (i.e., IL6, TNF-α), and chemokine levels, and thereby improving the symptoms of COVID-19 patients.

Omics approaches could be used to explain the complicated therapeutic mechanism of TCM against diseases from multiple levels (Xin et al., 2019; Guo et al., 2020), which could help us comprehend the relationship between TCM and human health. In the recent 20 years, high-throughput sequencing techniques have evolved at a breakneck pace. As omics technologies become mature, the multi-omics approaches are indispensable strategies for further understanding the therapeutic mechanism of TCM formulas (Buriani et al., 2012; Aronson and Rehm, 2015). In this review, we have summarized the omics approaches in TCM research, such as the prevalent chronic and metabolic diseases (i.e., diabetes), cancer (a leading cause of death worldwide, such as colorectal cancer, breast cancer, and lung cancer) (Fan et al., 2017; Lehmann et al., 2021; Sun et al., 2021), and infectious diseases (i.e., COVID-19, a global public health event) (Runfeng et al., 2020; Wu et al., 2021b; Chen et al., 2021), as well as the corresponding TCM formulas as examples to illustrate the critical role of multi-omics approaches for elucidating the therapeutic mechanism of TCM formulas in treating diseases (Figure 2).

### Intestinal microbiota-mediated anti-diabetic effects of berberine

Berberine as an extract of TCM formula from *Berberis*, *Coptis*, and *Hydrastis* (Habtemariam, 2020), is an excellent treatment for chronic and metabolic disorders diseases (Lan et al., 2015; Heindel et al., 2017). Its anti-diabetic function has been demonstrated by plenty of studies. Through metabolomics analysis, the researchers have found that berberine has improved glycometabolism (Dong et al., 2016; Yao et al., 2020) (Figure 2A). Zhang et al. (2010), used transcriptomics, proteomics, and found berberine increased the expression of InsR messenger RNA and protein in various human cells. They lowered the metabolites such as glycaemic levels, hemoglobin A, triglyceride levels, and increased insulin levels in type 2 diabetic (T2D) patients. As known, berberine exhibits poor oral bioavailability (Liu Chang-Shun et al., 2016a). While the intestinal microbes, which contain a specific enzyme called nitroreductase (NTR), are reported as the mediators that could convert berberine into absorbable molecule dihydroberberine (five times more absorption compared to berberine) for host, and then oxidize them back to berberine after absorption in the intestine (Chen et al., 2016; Feng et al., 2018) (Figure 2A). Zhang et al. (2020b), have used metagenomics and metabolomics data from the berberine treatment T2D patients, and found berberine reduced the glycaemic level by inhibiting deoxycholic acid species biotransformation by *Ruminococcus bromii*. Thus, the interactions between intestinal microbes and bioactive compounds of TCM formulas highlight the critical role of TCM and intestinal microbes in human health (Zhang et al., 2020a), implying that the intestinal microbes might be considered as potential targets for designing the drug delivery system.

### Multi-omics analysis for understanding the anti-cancer effects of *C*. *chinensis* Franch. (Ranunculaceae)

Based on the genomics approach (Figure 2B), Chen *et al.*, have used genomics approaches to analyze the genome of the *C*. *chinensis* Franch. (Ranunculaceae) and revealed the diversification of protoberberine-type alkaloids (Liu et al., 2021). They also discovered that the gene *cyp719* is involved in berberine biosynthesis through encoding the enzyme called (S)-canadine synthase. Through metabolome and transcriptome analysis, researchers have found the bioactive compounds of *C. chinensis* Franch. (Ranunculaceae) such as berberine could downregulate the expression of eukaryotic translation initiation factor 4A isoform 2 (*eif4a2*) gene (Lv et al., 2016; Chou et al., 2017; Wang et al., 2019) (Figure 2B), which indirectly emphasized the important role of *cyp719* gene of *C. chinensis* Franch. (Ranunculaceae) in anti-cancer effect. While *eif4a2* gene is considered a novel target for cancer therapy and anti-cancer drug discovery (Lv et al., 2016; Chou et al., 2017; Wang et al., 2019), its inhibition prevents the cooperation between *eif4a2* and oncogenes (Lindqvist and Pelletier, 2009), and thus effectively inhibits the replication of cancer and tumor (Lindqvist and Pelletier, 2009; Slaine et al., 2017).

### Multi-omics analysis for understanding the potential anti-infection effects of Lianhua Qingwen capsule

TCM formula may exert comprehensive influences, enhancing the host’s ability to resist viral infection (Yuan and Lin, 2000; Jiang, 2005) (Figure 2C). Among numerous infectious diseases, COVID-19 is a well-known global public health event, which brings trouble to our daily life and huge burden worldwide. Researchers have found numerous TCM formulas that may achieve therapeutic outcomes for COVID-19 patients through multiple pathological pathways and TCM-disease targets across stages (Ren et al., 2020; Wu et al., 2021b). Chai *et al.*, have analyzed the alteration of genetics, DNA methylation, and RNA expression for samples from COVID-19 patients, and found that during viral infection, angiotensin-converting enzyme 2 (ACE2) is identified as a membrane-binding receptor to the fusion and invasion process of the COVID-19 and host cells (Chai et al., 2020). And the COVID-19’ S protein (also called Spike protein; Figure 2C) could recognize and combine with the human cell protein ACE2, then use the host genetics to replicate new COVID-19 viruses (Hoffmann et al., 2020). These novel COVID-19 viruses were released and disseminated in the bloodstream. They then bind the ACE2 of other cells in COVID-19 patients, disrupting their equilibrium, and threatening other organs, such as the intestine, kidney, and heart of COVID-19 patients (Leung et al., 2020). Thus, the potential therapeutic effect of the TCM formula is to find the bioactive compounds to bind to host receptors such as ACE2, to prevent or inhibit the invasion/attachment of the virus to host cells (Figure 2C).

Lianhua Qingwen capsule is a classic patent TCM, which consists of 13 ingredients, including *Forsythia suspensa* (Thunb.) Vahl (Oleaceae; 255 g), *Lonicera japonica* Thunb. (Caprifoliaceae; 255 g), *Prunus armeniaca* L. (Rosaceae; 85 g), *Ephedra sinica* Stapf (Ephedraceae; 85 g), *Isatis indigotica* Fort. (Cruciferae; 255 g), *Dryopteris crassirhizoma* Nakai (Polypodiaceae; 255 g), *Rheum palmatum* L. (Polygonaceae; 51 g), *Houttuynia cordata* Thunb. (Saururaceae; 255 g), *Pogostemon cablin* (Blanco) Benth. (Blanco; 85 g), *Rhodiola crenulata* (Hook.f. and Thomson) H. Ohba (Crassulaceae; 85 g), *Mentha haplocalyx* Briq. (Lamiaceae; 7.5 g), *Gypsum Fibrosum* (255 g), *Glycyrrhiza uralensis* Fisch. ex DC. (Fabaceae; 85 g) (Xiao et al., 2020). This TCM is an innovative drug with potential therapeutic effects on respiratory diseases (Hu et al., 2022). Li *et al.*, have assessed the antiviral effectiveness of Lianhua Qingwen capsule against SARS-CoV-2 in Vero E6 cells and human hepatocellular carcinoma cells using cytopathic effect inhibition assay, plaque reduction assay, and real-time quantitative PCR analysis (Runfeng et al., 2020). They have found that TCM formula Lianhua Qingwen could block the replication and expression of proinflammatory cytokines (i.e., IL6, TNF-α). and chemokine (i.e., CXCL-10, IP-10) caused by COVID-19, and thus considerably alleviating the symptoms and the development of the COVID-19 in patients (Runfeng et al., 2020; Wu et al., 2021b) (Figure 2C). Chen *et al.* found 132 bioactive metabolites in Lianhua Qingwen capsule in treating COVID-19 (Chen et al., 2021). Among them, five compounds (forsythoside I, forsythoside A, rhein, neochlorogenic acid, and its isomers) were further identified with high inhibitory effects on ACE2 receptor of host cells, implying that they could inhibit the invasion of the virus to the host cells, and thus improved the symptoms of COVID-19 patients (Chen et al., 2021). Collectively, these efforts provided insight into the molecular mechanisms of therapeutic effects of Lianhua Qingwen capsule in treating COVID-19, thereby confirming the applicability and vital significance of the TCM formula in infectious illness treatment.

## Multi-omics approaches for TCM network analysis research

**FIGURE 3:**
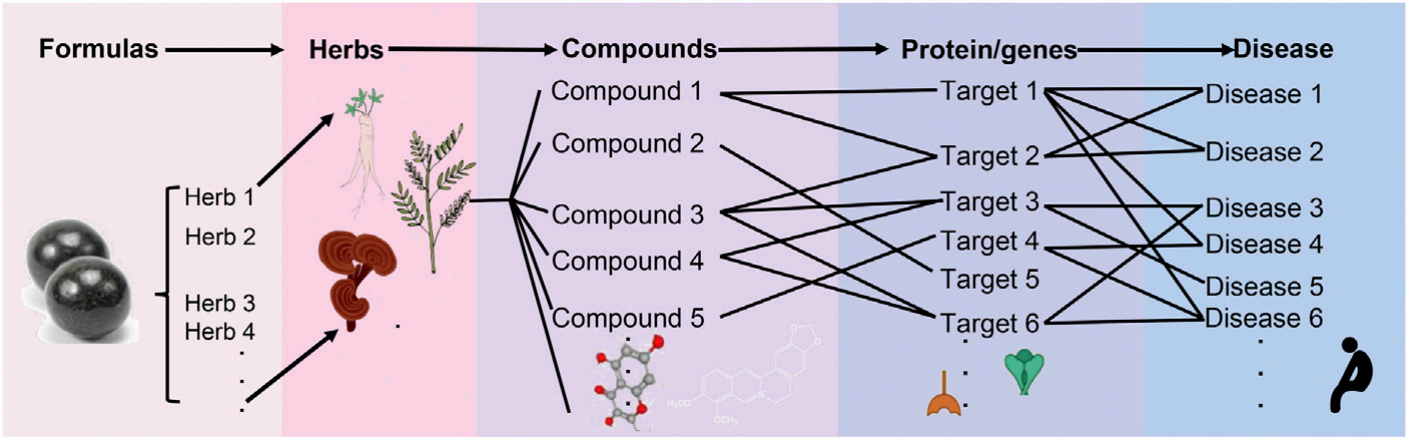
TCM network pharmacology analysis is proposed for leveraging the “ingredients-compounds-proteins/genes-diseases” network for TCM research. A TCM formula is made according to the Chinese Pharmacopoeia, herbal ingredients were the major prescribed ingredients in most TCM formulas. While based on network pharmacology analysis, chromatography, and high-throughput sequencing technology, we could decode the relationship between TCM formulas-species ingredients-compounds-targets-diseases. By looking at the bioactive compounds in the prescribed ingredients, we could learn more about the herb, the bioactive compounds, the target proteins, and the genes in treating diseases. This would help us understand how TCM works to treat different diseases.

One of the most successful applications of omics approaches is network pharmacology analysis. TCM formulas typically incorporate various ingredients and multi-targets for synergistic anti-disease effects (Figure 3), which coincides with the concept of network pharmacology analysis (Zhang et al., 2019b; Yang et al., 2022). Network pharmacology analysis is presented to clarify which compounds of TCM formula exert effects in treating diseases and which targets could be exploited for drug design (Boezio et al., 2017; Wu et al., 2018; Zhang et al., 2019b), which has developed into a powerful method for leveraging the “ingredients-compounds-proteins/genes-diseases” network for TCM research (Zhang et al., 2019b; Yang et al., 2022). Multi-omics such as transcriptomics, genomics, epigenomics, and proteomics could be used to determine the ingredients, proteins, and/or genes of TCM formulas, whereas electrochemical method, HPLC, real-time qPCR, and other quantified methods can be used to determine and quantify the content of bioactive compounds (Wolfender et al., 2003; Zhu et al., 2010; Wang et al., 2021). While network pharmacology analysis could facilitate the integration of ingredients, bioactive compounds, and proteins/genes into a network, providing an effective resolution for exploring the TCM-disease interaction (Zhang et al., 2019b).

### Network pharmacology analysis in decoding the roles of LDW in treating type 2 diabetes

T2D is a chronic metabolic disease that affects approximately affecting nearly 425 million people around global, leading to poor health status and massive economic outputs (Lin et al., 2015; Gan et al., 2019), causing substantial damage to the blood vessels, heart, and brain with long-time hyperglycemic status (Adeshara et al., 2016; Areosa Sastre et al., 2017). It was reported that Liuwei Dihuang Wan (LDW) is a patented TCM formula for treating T2D (Cheng et al., 2014; He et al., 2019). Based on the bioactive compounds recorded in TCMSP (30,069 bioactive compounds; http://sm.nwsuaf.edu.cn/lsp/tcmsp.php), BATMAN (25,210 bioactive compounds; http://bionet.ncpsb.org/batman-tcm/), and TCM Database@Taiwan (351 bioactive compounds; http://tcm.cmu.edu.tw/) databases, He et al. (2019), used the network pharmacology analysis and found 45 active compounds of LDW ingredients, which exert their pharmacological effects on T2D. As known, people with T2D were identified to have higher levels of inflammatory cytokines, such as IL-1B and IL-6, and lower levels of IL-10 (Kologrivova et al., 2014; Fathy et al., 2019), and the inflammatory cytokines disorder were also reported as biomarkers for early T2D diagnosis (Senthilkumar et al., 2018; Fathy et al., 2019). While the bioactive compounds of LDW, such as beta-sitosterol, isofucosterol, quercetin, are enriched in anti-inflammatory, anti-oxidative stress responses pathway by interacting with the target genes (i.e., *akt1*, *ptgs2*), which could decrease the inflammatory and remove oxidative stress and reactive oxygen species (Chasseaud, 1979; Sheehan et al., 2001), thus improved the symptoms in T2D patients. These findings imply that LDW may play an important role in treating T2D through the bioactive compounds interacting with the key or central genes in the development of T2D.

### Network pharmacology analysis in decoding the roles of TCM in treating the cardiovascular and cerebrovascular diseases

Cardiovascular and cerebrovascular disease (CCVD) is a term that refers to an illness that affects the heart, brain, or blood vessels (Tao et al., 2013; Wang et al., 2020). According to the WHO, this disease contributes to 32% of fatalities worldwide [https://www.who.int/news-room/fact-sheets/detail/cardiovascular-diseases-(cvds)]. Through network pharmacology analysis, Wang et al. (2020) have found and experimentally verified superoxide dismutase, glutathione peroxidase, malondialdehyde, and nitric oxide synthase of Safflower and Salvia as promising therapeutic targets for treating cerebral and myocardial infarction in. Safflower can decrease the cerebral infarction rate, cerebral cell edema, glial cell proliferation, and inflammatory cell infiltration, as well as increase the content of superoxide dismutase and glutathione peroxidase, and decrease the content of malondialdehyde and nitric oxide synthase (Wang et al., 2020). Moreover, Tao et al. (2013), incorporated the chemical compounds including the chemical structure and pharmacological information, as well as biological functional data of TCM formula *Radix Curcumae*, and found 58 bioactive compounds and 32 potential targets for the prevention and/or treatment of CCVD. In summary, network pharmacology analysis sheds fresh light on the TCM-disease interactions, providing potential therapeutic targets for drug discovery.

### Network pharmacology analysis in decoding the roles of TCM in treating cancer

With the advancement of multi-omics approaches, many TCM formulas have been mined for cancer prevention and treatment (Fan et al., 2017; Xiang et al., 2019; Lehmann et al., 2021; Sun et al., 2021). In this review, we took CRC as an example to elucidate the omics approaches underlying the mechanisms of TCM against cancer. CRC is diagnosed as the third major malignant tumor worldwide, which contributes to the fourth leading cause of cancer death (Arnold et al., 2017). Using network analysis to integrate multiple-omics data, researchers have found that the bioactive compounds in the TCM formula Huang Lian-Gan Jiang (Gingerenone C, Obaculactone, and Isogingerenone B) could interact with the main targets of CRC (i.e., PDE5A, TGFBR2, HRAS) to regulate the signaling pathways (i.e., DAP12, NGF, AKT signaling pathway), contributing to therapeutic effects for CRC patients (Gong et al., 2019). Among these targets, FGFR4 has been confirmed with the ability to inhabit CRC cell proliferation, promote apoptosis, and disrupt the cell cycle, which could be a novel target for drug design and new therapeutic direction (Jiang et al., 2017). In addition, Shaoyao decoction was verified to increase the survival rate and decrease the occurrence of colonic neoplasms in the mice model (Lin et al., 2014). Moreover, through network pharmacology analysis, the bioactivate compounds Zuojinwan: quercetin, baicalein, and wogonin combined with the target genes *akt1*, *jun*, and thus activate the important pathway such as PI3K-Akt signaling pathway to induce apoptosis to exert anti-cancer effects in CRC patients (Huang et al., 2020). Relevant scientific research also demonstrated that the *Ampelopsis ethanolic* extract could inhibit *stat3* and Src phosphorylation, and downregulate the expression of their target genes, such as *bcl-xL*, *mmp*-2 in CRC patients (Sun et al., 2021). These curative or preventative effects of TCM for various diseases have been demonstrated in rodent or human studies (Sun et al., 2021). The candidate bioactive compounds and targets screened by network pharmacology analysis could guide new drug discovery and therapeutic direction.

## Databases and analytical resources for TCM research

The TCM-associated databases are repositories for storing omics data, including ingredients, compounds, genes, targets, and diseases. Based on a comprehensive screening analysis of TCM-associated databases, the network pharmacology analysis has further deepened the therapeutic effects of TCM. Numerous efforts have also been put into the establishment of TCM-associated databases. In this section, we present the most frequently used databases, and then use the well-studied ingredients to assess the properties of these TCM-associated databases.

### Current TCM-associated database for TCM research

**FIGURE 4:**
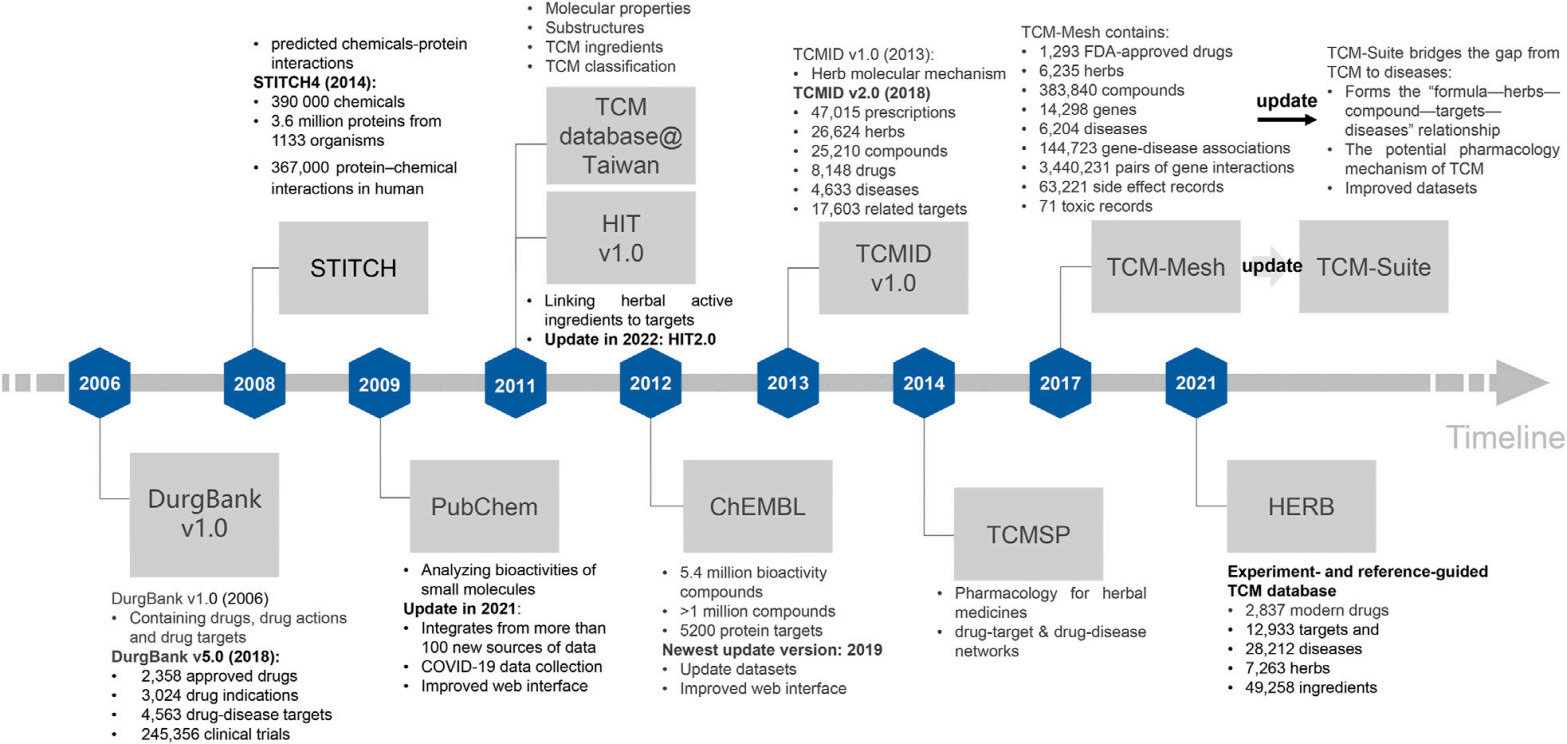
The development of commonly used TCM-disease databases along the timeline. We described the TCM databases based on their initial and most recent updated versions, respectively, which highlighted the primary composition, function, and characteristics of these databases along the timeline. The updated version of a database is highlighted in bold font of the description.

Currently, numerous TCM databases have emerged in response to the need for different aspects of TCM-disease studies (Mendez et al., 2019; Fang et al., 2021). In this review, we processed the development of the most frequently used TCM databases and their updated versions during our evaluation (Figure 4).

DurgBank (http://www.drugbank.ca) was initially reported in 2006, and has been updated continuously over the past years (Wishart David S. et al., 2006). The most recent version is DurgBank 5.0, which comprises 2,358 FDA-approved small molecular pharmaceuticals and 4,964 experimental drugs, as well as 7,129 unique drug targets and other drug-associated informantion (Wishart et al., 2018) (Figure 4). STITCH is primarily concerned with decoding chemical-protein interactions (Kuhn et al., 2008), its most recent version STITCH4 has 390,000 chemicals and 3.6 million proteins from 1,133 organisms, and 367,000 protein–chemical interactions in human (Kuhn et al., 2014). PubChem (http://pubchem.ncbi.nlm.nih.gov) is used to search for the 2D and 3D bioactivities of small molecules (Wang et al., 2009), which was updated in 2021. The updated version of PubChem has integrated more than 100 Corona Virus Disease 2019 (COVID-19) datasets, such as chemical, physical property, and patent associated information, and supports various types of structure search (such as identity, similarity, substructure, and superstructure searches) *via* its web interfaces (Kim et al., 2021) (Figure 4). TCM Database@Taiwan (http://tcm.cmu.edu.tw/) is one of the largest non-commercial TCM databases for drug screening that launched in 2011 and contains over 20,000 pure compounds derived from 453 TCM ingredients (Chen, 2011). HIT (Herb Ingredients’ Targets, http://lifecenter.sgst.cn/hit/) is a database that connects herbal active ingredients to targets (Ye et al., 2011), which is updated in 2022 (HIT2.0). HIT2.0 has 10,031 compound-target activity pairs with quality indicators between 2,208 targets and 1,237 ingredients from more than 1,250 reputable herbal materials (Yan et al., 2022). ChEMBL (https://www.ebi.ac.uk/chembldb) contains 5.4 million bioactivity compounds for more than 1 million compounds and 5,200 protein targets (Mendez et al., 2019), and the most recent upgraded version is in 2019 with the improved dataset and web interfaces (Mendez et al., 2019). TCMID database (Traditional Chinese Medicine Integrated Database, http://www.megabionet.org/tcmid/) integrates the molecular mechanism of herbal materials (Xue et al., 2013). Its updated version TCMID2.0 is more comprehensive and contains 18,203 herbal ingredients, 15 prescriptions, 82 associated targets, 1,356 drugs, 842 diseases, and 170 herbal materials associated with mass spectrometry spectra (Huang et al., 2018). TCMSP (http://sm.nwsuaf.edu.cn/lsp/tcmsp.php) has considered the pharmacodynamics and pharmacokinetics for systemic pharmacology research, such as the bioactive compounds and compound-related targets screening (Ru et al., 2014), which includes 499 herbal materials with 29,384 ingredients, 3,311 targets, and 837 associated diseases in ChP, as well as 12 important absorptions, distribution, metabolism, and excretion related properties (Ru et al., 2014). TCM-Mesh (http://mesh.tcm.microbioinformatics.org/) was established based on the concept of the “TCM formula-compounds-proteins/genes-diseases” network to elucidate the regulatory mechanisms governing tiny molecules in TCM formula, as well as recording their side-effects (Zhang et al., 2017). Since pharmacotranscriptomics has become a powerful method for assessing the therapeutic efficacy of drugs, and identifying novel drug targets, HERB (high-throughput experiment- and reference-guided database of TCM, http://herb.ac.cn/) is built based on manually curated 1,241 gene targets and 494 modern diseases for 473 herbal materials, linking with 12,933 targets and 28,212 diseases to 7,263 herbs and 49,258 ingredients (Fang et al., 2021). To solve the problem of lacking holistic and systematical analysis of TCM formula, the newest database named TCM-suite (http://tcm-suite.aimicrobiome.cn/) was built (Yang et al., 2022). This database is a holistic pipeline that connects TCM biological and chemical ingredient identification and downstream network pharmacology analysis. It establishes a detailed “TCM formula-ingredients-compounds-proteins/genes-diseases” relationship. It enables users to identify components of a TCM formula and investigate its potential pharmacology mechanism and side effects simultaneously. These databases with different usages have provided the opportunity to explore the potential pharmacology mechanism of TCM formula at the molecular level in the treatment of numerous diseases.

### Comparing the properties of frequently used TCM database

The frequently used TCM databases also have different focuses and comprehensiveness. In this work, the databases (Google Scholar citations: as of April 2022: TCMSP: 1748, TCMID: 580, TCM-Mesh: 114), as well as TCM-Suite (http://tcm-suite.aimicrobiome.cn/) were chosen for comparison of their properties (Table 2). By comparing the results from different databases, we found that although the bioactive compounds recorded in the TCM-Suite database were fewer than those in other TCM-associated databases, the data of compound-target associations and target-disease associations were more abundant in this database. Additionally, we have discovered TCM-Suite database is more comprehensive in terms of the compound-target associations and target-disease associations. Most of the ingredients, with fewer bioactive compounds being collected in this database, were matched with more hits in bioactive compound-target associations and target-disease associations. The fewer bioactive compounds in TCM-Suite are probably because the TCM-Suite database removes the ambiguous bioactive compounds of TCM formulas.

**TABLE 2:** Comparing the properties of four representative TCM databases based on the selected ingredients reported in the previous study (Zhang R. et al., 2019b).

Ingredients	TCMID			TCMSP			TCM-mesh			TCM-suite		
	C	C-T	T-D	C	C-T	T-D	C	C-T	T-D	C	C-T	T-D
*Ganoderma lucidum* (Leyss. ex Fr.) Karst (Polyporaceae)	160	1,164	771	32	150	238	484	543	365	40	1,477	9,860
*Panax ginseng* C. A. Mey (Araliaceae)	293	4,820	2,913	153	4,144	2,970	380	748	486	134	7,045	19,400
*Codonopsis pilosula* (Franch.) Nannf. (Campanulaceae)	187	2,531	1,631	104	2,493	2,211	268	911	556	97	4,158	12,373
*Astragalus membranaceus* Bunge. (Fabaceae)	70	1,832	1,186	35	2,426	2,376	174	953	605	53	5,737	12,463
*Dioscorea oppositifolia* L. (Dioscoreaceae)	42	1,004	655	20	959	1,307	142	851	451	20	1,722	10,726
*Panax notoginseng* (Burk.) F.H.Chen (Araliaceae)	157	900	635	71	1,104	1,591	238	716	506	2	3	24
*Polygonum multiflorum* Thunb. (Polygonaceae)	73	618	599	—	—	—	24	513	888	14	1,155	7,728
Radix *Angelicae dahuricae* (Hoffm.) Benth. and Hook.f. ex Franch. and Sav. (Apiaceae)	135	406	289	446	1,408	454	80	362	477	7	107	1,947
*C. chinensis* Franch*.* (Ranunculaceae)	30	238	231	96	596	513	23	142	274	33	453	5,261
*Cordyceps sinensis* (BerK.) Sacc. (Ophiocordycipitaceae)	37	1,010	519	76	369	483	21	919	944	37	2,169	8,663

Notes, the numbers in the table indicate the search results in the corresponding databases. C: the number of the bioactive compounds; C-T: the number of compound-target associations; T-D: the number of target-disease associations. Though TCM-Suite (http://tcm-suite.aimicrobiome.cn/) is the newest version of TCM-Mesh, this database is a comprehensive and holistic platform for TCM composition identification and network pharmacology analysis. For the first time, this database constructed a holistic pipeline to interconnect TCM biological ingredient identification and downstream network pharmacology analysis, which also allows users to identify components of a TCM formula and investigate its potential pharmacology mechanism. The names of these ingredients were recorded in Chinese Pharmacopoeia (ChP) and/or validated taxonomically in http://mpns.kew.org/mpns-portal/or http://www.plantsoftheworldonline.org (Rivera et al., 2014).

## Discussion

**FIGURE 5:**
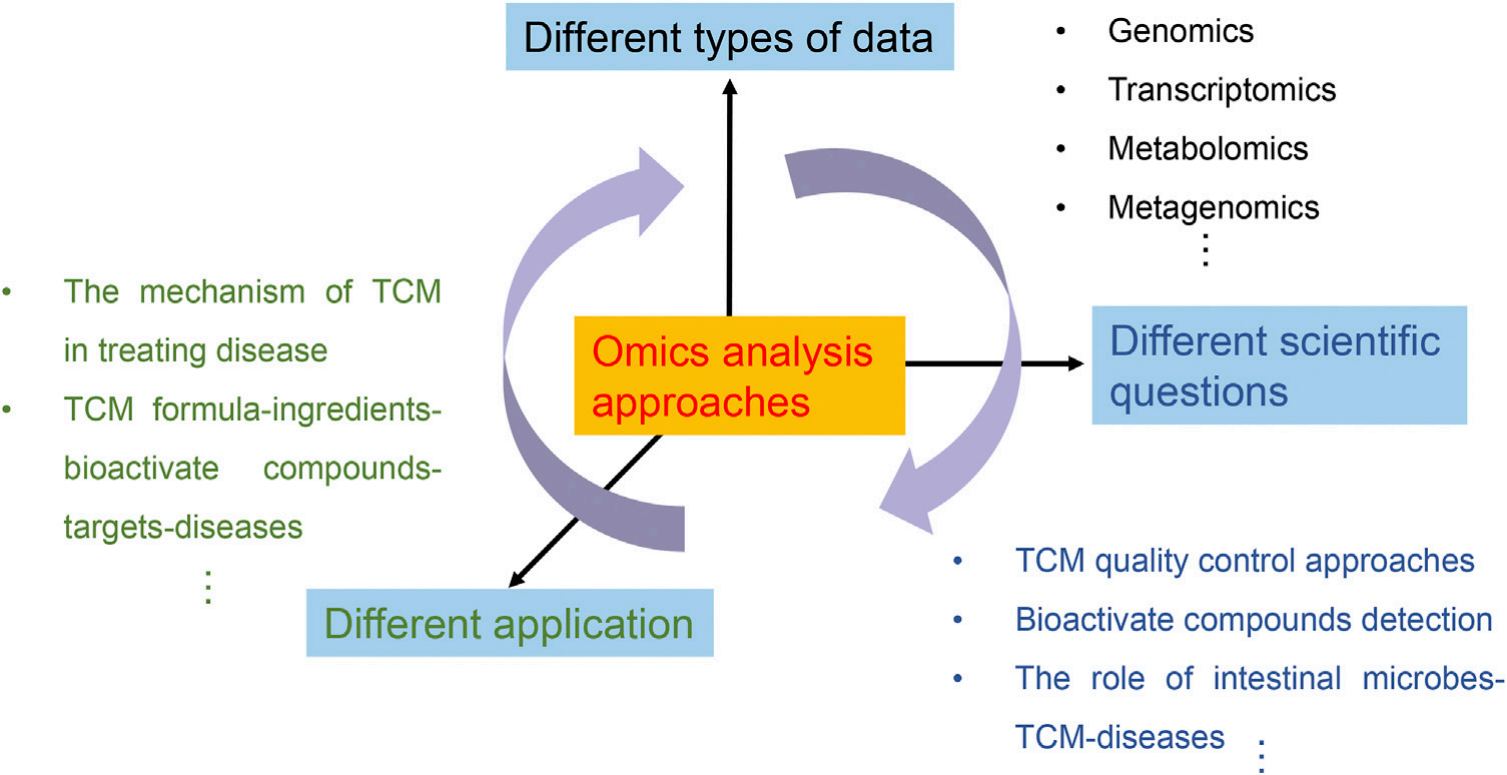
TCM research in the omics age has utilized different types of omics data to address different scientific questions, and meet the needs of different applications. With the development of sequencing technology, different types of data including genomics, transcriptomics, metabolomics, and metagenomics data could be gathered to address various scientific topics. The molecular mechanism of TCM formula in treating various diseases is gradually exposed by omics analysis approaches, and the relationship of TCM bioactive compounds-targets-diseases is constructed, providing suggestions for drug-target design. These scientific questions in turn contribute to the practical value of TCM materials, and the different applications will also create different types of data. This bidirectional interaction reinforces the clinical use of TCM and drives its modernization.

While numerous works have evaluated the multi-omics approaches in TCM research, this is the first systematic and comprehensive review of omics approaches in TCM research from a holistic standpoint. In this review, we have summarized the recent progress of multi-omics approaches in deciphering the mechanism of TCM against various diseases as compared to the previous research and discussed the advantages and limitations, as well as the future direction of multi-omics applied to the field of TCM. With the development of systems biology-driven omics analysis, TCM research has profoundly advanced (Wu et al., 2019; Wu al., 2021a; Gao et al., 2021), which means that TCM studies have already undergone a paradigm shift from the traditional biochemical and molecular analysis approaches to the data-driven omics analysis approaches (Figure 5). Analytical chemistry and biology approaches have pushed forward the development of TCM research and laid the groundwork for applying omics approaches (Zeng et al., 2019). In contrast, the omics approaches have the potential to significantly supplement the traditional analysis approach with more precise and comprehensive approaches, which has undoubtedly made a significant contribution to deciphering the mechanism of TCM from various aspects (bioactive compounds, TCM-drug targets, pharmacodynamic effects, and therapeutic mechanisms). This accomplishment has also sparked various anxieties and concerns for TCM, given the volume of heterogeneous data on issues that may be solved using omics data for the TCM research areas (Figure 5).

In this review, we have evaluated the TCM formulas from four aspects to address several anxieties and concerns about TCM. Firstly, the inconsistent quality control methods make TCM disputable (Yao Q. et al., 2022). Based on the advancement of sequencing and chromatographic technologies, researchers have combined chemical methods (i.e., HPLC, TLC) (Wolfender et al., 2003; Zhu et al., 2010; Wang Binbin et al., 2021) and biological methods (DNA fragments: ITS2, *trnL*, *trnH-psbA*, and COI) (Vences et al., 2005; Jia et al., 2017; Xin et al., 2018a; Xin Tianyi et al., 2018b; Zhang et al., 2019a; Chandrasekara et al., 2021) for quality control of TCM formula through detecting their prescribed ingredients. These initiatives have also yielded significant results in terms of evaluating the quality control of TCM formulas^18-20^, which would push forward the digital management of TCM. These advancements are inseparable from the database with the storage of referenced materials and the omics approaches, which ensure the legality and therapeutic efficiency of TCM formulas (Newmaster et al., 2006; Chen et al., 2014; Cheng et al., 2014; Guo et al., 2020; Subramanian et al., 2020; Besse et al., 2021).

Secondly, omics analysis approaches would provide clues for clinical applications of TCM formulas, thereby deepening our understanding of the therapeutic mechanism of TCM formulas. Numerous forms of data for TCM research have been analyzed to understand the therapeutic effects of TCM formulas on human diseases (Wang et al., 2015; Dong et al., 2016; Subramanian et al., 2020). Because the majority of the TCM formulas are administered orally, which typically come into contact with intestinal microbes and are transformed into host absorbable molecules under microbes, allowing for optimal utilization of TCM formulas (Feng et al., 2015; Chen Feng et al., 2016; Feng et al., 2018). Through the omics approach, TCM formulas have also been applied in the treatment of certain diseases, and show outstanding anti-diseases, such as cancer (Xiang et al., 2019; Liu et al., 2020), COVID-19 (Fan et al., 2020; Leung et al., 2020), and chronic diseases (Deng, 2010; Dong et al., 2016; Yang et al., 2019; Guo et al., 2020). All of these findings, which are based on omics datasets and approaches, have allowed for the identification of the mechanism underlying significant therapeutic effects (Feng et al., 2015; Liu et al., 2016a; Chen et al., 2016; Feng et al., 2018; Zhang et al., 2020a).

Thirdly, with its notion of integrity, comprehensiveness, and systematic approach, network analysis offers new strategies and approaches for researching TCM formulas, as it may transform TCM from an experience-based to an evidence-based medical system. From a network view based on different types of omics datasets and approaches, we can decode the molecular mechanism of the complicated TCM formulas (Tao et al., 2013; Li et al., 2014). That is, the relationship between TCM formulas and diseases could be divided into prescribed ingredients, bioactive compounds of prescribed ingredients, compounds-targets, and diseases-targets interactions (Zhang et al., 2017), which confirms the advantages of multi-compound, multi-target and multi-pathway characteristics of TCM formula and providing a reliable solution for systemic analyzing the clinical efficacy of TCM formula (Luo et al., 2020). These efforts will benefit target discovery, bioactive compound screening, toxicity evaluation, and the mechanism elucidation of TCM. These efforts may deepen our understanding of how TCM formulas could treat diseases. Based on these advantages, future applications of multi-omics approach in TCM research could be focused on drug mining, drug design, drug re-purposing, and therapeutic mechanisms investigation to make contributions to the modernization of TCM. Network pharmacology analysis is a systems biology-based approach, which selects specific signal nodes for multi-target drug molecule design to uncover the molecular mechanisms underlying the pharmacodynamic effects (Luo et al., 2020). This analytical approach largely depends on the high volume of data, while the current resources including medicines, genes, proteins, *etc.* are not exhaustive (Yang et al., 2022). It cannot be assured that the databases on TCM, bioactive compounds of TCM, and TCM-related gene targets are accurate and comprehensive. Moreover, no suitable calculation methods have been developed to calculate the correlations in network analysis. Network pharmacology analysis employs computer network screening for target selection, and these correlations were calculated without considering the distribution character of TCM data. Hence, influenced by cascade amplification, the number of disease targets and medication target proteins is gradually expanding, and the interpretation of data cannot keep up with the growth of data. Additionally, the network pharmacology analysis in TCM, such as new targets, or drug mechanism discovery, is still qualitative, and the scientific and mechanism discovery of TCM through network analysis still needs critical and extensive clinical trials. Furthermore, the dose-efficacy relationship between the TCM formula and disease remains further explored and quantified. Last but not least, human disease is dynamic changes, so the course of disease development and treatment efficacy process should be considered in further studies.

Fourthly, the TCM-associated databases provide plenty of TCM-related data, which were created and are continuously updated with the demand for TCM research. We proposed the TCM-Suite database (http://tcm-suite.aimicrobiome.cn/) (Yang et al., 2022), which overcomes the limitations of the currently available databases for network analysis and other omics analysis, and serves as a bridge for integrating the prescribed ingredients of TCM formulas, the bioactive compounds of prescribed ingredients, compounds-targets, and disease-targets as a holistic concept. This database establishes the foundation for the systemic investigation of the therapeutic effects from a network analysis perspective, facilitating the clinical application value and drug development. Given the importance of intestinal microbes in assisting the host’s absorption of bioactive compounds (Feng et al., 2015; Chen et al., 2016; Feng et al., 2018), further database developments should take the interaction between intestinal microbes, TCM formulas, and human symptoms into consideration for building up more complete and exhaustive databases, making the TCM research more concise and convenient. With the need for TCM research, various TCM-related databases have emerged, but the query of these databases is quite different. For example, the herbal material “*C*. *chinensis* Franch.” could be accessed by searching “huang lian” or “*Coptidis*” in TCMID (Huang et al., 2018), “huanglian” in TCMSP (Ru et al., 2014), whereas in the TCM-Suite database (Yang et al., 2022), this herbal material could be accessed by searching “huang lian” or “C. *chinensis*.” And thus, a platform considering the various official name/ID or a standard platform for TCM research is needed, which could make the databases more concise and available, enabling the researchers to make full use of the resources.

Collectively, systems biology and data-driven omics analysis approaches, including genomics, proteomics, metagenomics, transcriptomics, and metabolomics, play an indispensable role in decoding the interactions of TCM and diseases, which could comprehensively and deeply decode the therapeutic mechanisms of the TCM formula. These efforts have hastened the translation of TCM from fundamental research to a broad spectrum of applications, particularly in clinical applications.

However, apart from the aforementioned limitations for each part of TCM analyses, there are also several limitations of the multi-omics approach applied to TCM. Consider network analyses is still at a very preliminary level of evidence and might often be used misleadingly. Firstly, the high dimensionality, high complexity, and high volume of heterogeneous data make it difficult for TCM analysis. Secondly, there is a lack of data resources for in-depth interpretation of TCM preparations: from identifying herbal ingredients and their bioactive compounds to understanding how small molecules in herbal formulas interact with genes and proteins in the human body. And the high chemical complexity of TCM makes it challenging to characterize their bioactive compounds and investigate systemic actions in humans. Thirdly, the limited knowledge of biochemistry has constrained our further understanding of TCM pharmacological and toxicological effects. Fourthly, even under the idea of “homology of medicine and food” (Hou and Jiang, 2013; Gong et al., 2020), there is limited knowledge about the medicinal value and mechanism of medicinal and edible plants and their effects on human health. A deeper understanding of these medical homologous plants will help people adjust their health according to their daily diet. Fifthly, even though multi-omics have been extensively used in TCM research and clinical practices, more digital information, including protein structure information, biological images, as well as electronic medical records, could be integrated for better understanding of the TCM-disease relationships. Finally, the application of omics approaches in new drug discovery still has a long way to go, pharmacodynamic effects of the new drugs should perform with critical clinical trials. In the future research, it will be useful to highlight the current pitfalls in these approaches and how they can be overcome.

To achieve this, a bioinformatician with good clinical training should act as a bridge to translate multi-omics approaches and datasets into routine clinical practice to assist with clinical diagnosis. We believe these efforts are beneficial for the process of TCM towards modernization, internationalization, and digitalization in the treatment of a variety of complicated diseases.

## Conclusions

In this review, we have summarized the recent progress of multi-omics approaches in deciphering the mechanism of TCM against various diseases as compared to the previous research and discussed the advantages and limitations, as well as the future direction of multi-omics applied to the field of TCM. With the development of systems biology-driven omics analysis. And we suggested combing the chemical and biological ingredients analytical methods to get a more objective and comprehensive assessment of TCM quality. Moreover, with the help of bioinformaticians, omics analysis approaches would accelerate the unveiling of the mystery of TCM, and move it towards internationalization. Besides, assisted by network analysis, the link between TCM and diseases could be visualized from ingredients to compounds to targets, which could enhance our understanding of the relationship between TCM and diseases. Furthermore, with heterogeneous TCM data, databases serve as a repository for these data, which promote the modernization and internationalization of TCM and potentially provide critical technological support for clinical diagnosis, drug development, and precision medicine. We believe under the guidance of bioinformaticians, these efforts are advantageous for the TCM process including modernization, internationalization, and digitalization.
